# Migration of the contractile phenotype of human airway smooth muscle cells in response to supernatants from rhinovirus infected human bronchial epithelial cells

**DOI:** 10.1186/1710-1492-10-S1-A44

**Published:** 2014-03-03

**Authors:** Abid Qureshi, Sami Shariff, Sergei Nikitenko, Jason Arnason, Chris Shelfoon, Suzanne Traves, David Proud, Richard Leigh

**Affiliations:** 1 Snyder Institute for Chronic Diseases, University of Calgary, Calgary, Alberta, T2N 4N1, Canada

## Rationale

Human rhinovirus (HRV) infections during early childhood are associated with a significantly increased risk of developing asthma in subsequent years [1]. There is published evidence that airway remodeling is present in pre-school children, often before the diagnosis of asthma is established [1]. It is thought that this increased risk relates to the fact that HRV infections facilitate airway remodeling in asthma [2]. A feature of airway remodeling is the proximity of airway smooth muscle (ASM) to the subepithelial region among other pathological changes [3]. Smooth muscle is also known to exist in two distinct phenotypes: secretory and contractile [4]. We have recently shown that HRV infection of Human Bronchial Epithelial Cells (HBEC), both *in vitro* and *in vivo*, results in the up-regulation of a number of airway remodeling mediators [5]. We now sought to determine which ASM phenotype (contractile or secretory) results in migration to supernatants from HRV infected HBE cells.

## Methods

Primary HBE cells were cultured in growth medium until confluent, pre-treated with 1% insulin, transferrin, and selenium (ITS) medium for 24 hours and then stimulated with media-control or purified HRV-16. The ASM D9 cell-line was obtained from Dr. Andrew Halayko’s laboratory and cultured in T-175 flasks in 10% serum containing Dulbecco's Modified Eagle Medium (DMEM; Gibco; secretory phenotype) or 1% ITS F-12 media (Gibco; contractile) until they reach ~90-100% confluence. HBEC supernatants were used as chemo-attractants for ASM (4hrs) migration through 8 μm pore polycarbonate filter in a 48-well Boyden Chamber. Migrated cells on filter were fixed/stained via Diff-quick and counted at 200x view.

## Results

ASM D9 cells treated with 1% ITS F12 media showed significantly higher levels of migration to fetal bovine serum (FBS) compared to ASM D9 cells treated with serum (n=3, p < 0.001). Preliminary data indicate that HRV-16 infected HBEC supernatants resulted in greater ASM migration compared to HBEC supernatant from media alone in the ASM D9 contractile phenotype but not the secretory phenotype.

## Conclusions

These findings support our hypothesis that the contractile phenotype of ASM D9 cells, which is more representative of ASM cells *in vivo*, migrate better than the secretory phenotype. Additionally, strong preliminary data indicate that supernatants from HRV infected HBE cells promotes ASM chemotaxis, and provides additional evidence for the role of contractile ASM, and HRV infections, in the pathogenesis of airway remodeling. Ongoing studies will examine protein expression between the two different phenotypes.
